# Polymorphisms in the Toll-Like Receptor and the IL-23/IL-17 Pathways Were Associated with Susceptibility to Inflammatory Bowel Disease in a Danish Cohort

**DOI:** 10.1371/journal.pone.0145302

**Published:** 2015-12-23

**Authors:** Steffen Bank, Paal Skytt Andersen, Johan Burisch, Natalia Pedersen, Stine Roug, Julied Galsgaard, Stine Ydegaard Turino, Jacob Broder Brodersen, Shaista Rashid, Britt Kaiser Rasmussen, Sara Avlund, Thomas Bastholm Olesen, Hans Jürgen Hoffmann, Bjørn Andersen Nexø, Jacob Sode, Ulla Vogel, Vibeke Andersen

**Affiliations:** 1 Medical Department, Viborg Regional Hospital, Viborg, Denmark; 2 Biomedicine, University of Aarhus, Aarhus, Denmark; 3 Microbiology and Infection Control, Statens Serum Institut, Copenhagen, Denmark; 4 Veterinary Disease Biology, University of Copenhagen, Copenhagen, Denmark; 5 Department of Gastroenterology, Herlev Hospital, Herlev, Denmark; 6 Department of Gastroenterology, Hvidovre Hospital, Hvidovre, Denmark; 7 Medical Department, Køge Hospital, Køge, Denmark; 8 Medical Department, Hillerød Hospital, Hillerød, Denmark; 9 Medical Department, Sydvestjysk Hospital, Esbjerg, Denmark; 10 Department of medical Gastroenterology, Odense University Hospital, Odense, Denmark; 11 Medical Department, Bispebjerg Hospital, Bispebjerg, Denmark; 12 Medical Department, Nykøbing Falster Hospital, Nykøbing Falster, Denmark; 13 Medical Department V, Aarhus University Hospital, Aarhus, Denmark; 14 Medical Department, Slagelse Hospital, Slagelse, Denmark; 15 Department of Respiratory Diseases B, Institute for Clinical Medicine, Aarhus University Hospital, Aarhus, Denmark; 16 Institute of Regional Health Research, University of Southern Denmark, Odense, Denmark; 17 Department of Autoimmunology and Biomarkers, Statens Serum Institut, Copenhagen, Denmark; 18 Department of Rheumatology, Frederiksberg Hospital, Frederiksberg, Denmark; 19 National Research Centre for the Working Environment, Copenhagen, Denmark; 20 Research unit for Molecular Diagnostic, Hospital of Southern Jutland Aabenraa, Aabenraa, Denmark; 21 OPEN Odense Patient data Explorative Network, Odense University Hospital, Odense, Denmark; INSERM, FRANCE

## Abstract

**Background:**

The inflammatory bowel diseases (IBD), Crohn’s disease (CD) and ulcerative colitis (UC), result from the combined effects of susceptibility genes and environmental factors. Previous studies have shown that polymorphisms in the Toll-like receptor (TLR), the apoptosis, the IL-23/IL-17 and the interferon gamma (IFNG) pathways are associated with risk of both CD and UC.

**Methods:**

Using a candidate gene approach, 21 functional single nucleotide polymorphisms (SNPs) in 15 genes were assessed in a clinical homogeneous group of severely diseased ethnic Danish patients consisting of 624 patients with CD, 411 patients with UC and 795 controls. The results were analysed using logistic regression.

**Results:**

The polymorphisms *TLR5* (rs5744174) and *IL12B* (rs6887695) were associated with risk of CD, and *TLR1* (rs4833095) and *IL18* (rs187238) were associated with risk of both CD and UC (p<0.05). After Bonferroni correction for multiple testing, the homozygous variant genotype of *TLR1* 743 T>C (rs4833095) was associated with increased risk CD (OR: 3.15, 95% CI: 1.59–6.26, p = 0.02) and CD and UC combined (OR: 2.96, 95% CI: 1.64–5.32, p = 0.005).

**Conclusion:**

Our results suggest that genetically determined high activity of *TLR1* and *TLR5* was associated with increased risk of both CD and UC and CD, respectively. This supports that the host microbial composition or environmental factors in the gut are involved in risk of IBD. Furthermore, genetically determined high activity of the IL-23/IL-17 pathway was associated with increased risk of CD and UC. Overall, our results support that genetically determined high inflammatory response was associated with increased risk of both CD and UC.

## Introduction

Chronic inflammatory bowel diseases (IBD), Crohn’s disease (CD) and ulcerative colitis (UC), are complex diseases that result from the interaction of numerous genetic and environmental factors [1]. Genetic association studies have identified polymorphisms in the Toll-like receptor (TLR) [2,3], the apoptosis [4–6], the IL-23/IL-17 [4–6] and the interferon gamma (IFNG) [4–6] pathways associated with susceptibility of CD and UC. As described below the inflammasome is connected to all of these pathways and it could be speculated that polymorphisms in the inflammasome pathway could be associated with CD or UC.

These pathways are involved in the autoimmune response to Pathogen-Associated Molecular Patterns (PAMPs) such as bacterial or viral DNA, flagellin or lipopolysaccharide (LPS). PAMPs can be bound by the membrane bound TLRs and induce inflammation by activating a number of pro- and anti-inflammatory cytokines [7]. PAMPs can also be recognized by Nod-like receptors (NLRs), which are intracellular receptors. NLRP1 and NLRP3 are members of the inflammasome protein complexes, which can activate the pro-protein caspase-1. The activation of caspase-1 can be suppressed by CARD8 [8]. Caspase-1 activates the pro-inflammatory cytokines IL-1β and IL-18, which are synthesized as pro-proteins [9]. In turn, IL-1β and IL-18 can induce the production of IL-17 [10].

NLRP1, NLRP3 and CARD8 have also been described to be involved in the apoptotic pathway, where overexpression of NLRP1 and NLRP3 stimulates apoptosis through activation of caspases [11,12] and CARD8 inhibit apoptosis by inhibiting the activation of caspase-1 [13].

In the IL-23/IL-17 pathway, IL-23 enhances the secretion of the pro-inflammatory cytokine IL-17, which in turn enhances the production of pro-inflammatory mediators such as IL-1β, IL-6, IL-8 and TNF-α [14]. IL-23 is a heterodimer composed of IL-12p40 and IL-23p19. IL-12p40 also acts as a subunit of the IL-12p35/IL-12p40 heterodimer cytokine IL-12. IL-12 can act pro-inflammatory by activating IFN-γ and anti-inflammatory by activating the anti-inflammatory cytokine IL-10 [15] and by inhibiting the pro-inflammatory cytokine IL-17 [14]. The IL-12 and IL-23 pathways also share the receptor subunit IL12R-β1, which is part of the IL12 receptor (IL12R-β1/IL12R-β2) and IL23 receptor (IL12R-β1/IL23R) heterodimer complex. The signalling through the IL12 and IL23 receptor also share intracellular mechanisms, where both receptors bind the Jak2 kinase, which in turn activates the STAT4 transcription factor. However, the resulting DNA binding STAT transcription complexes differ and involve the STAT3/STAT4 heterodimer in IL23 signalling rather than the STAT4 homodimer in IL12 signalling [14].

IL-18, which can be activated by the inflammasome, can feedback activate the synthesis of IFN-γ. IFN-γ binds to the membrane bound IFNG receptor which is comprised of two ligand-binding IFNGR1 chains and two signal-transducing IFNGR2 chains. Binding of IFN-γ to the IFNG receptor recruits the Jak2 kinase. Jak2 initiates a kinase cascade, which ultimately activates the transcription factor STAT1 [16]. Another important activator of IFN-γ is the transcription factor T-bet (*TBX21*) [17].

In this study, we wanted to investigate whether functional single nucleotide polymorphisms (SNPs) in genes involved in the Toll-like receptor (*TLR1*, *TLR5* and *TIRAP*), the inflammasome or apoptotic (*NLRP1*, *NLRP3* and *CARD8*), the IL-23/IL-17 (*IL12B*, *IL12RB1*, *IL12RB2*, *IL17A*, *IL18* and *JAK2*) and the IFNG (*IFNGR1*, *IFNGR2* and *TBX21*) pathways were associated with risk of CD or UC in a Danish cohort of severely diseased patients. Knowing the biological effects of the studied polymorphisms allows a biological interpretation of the associations based on increased or decreased gene activity [3,18,19].

## Materials and Methods

### Cohort

As described by Bank et al. an ethnic Danish cohort consisting of 624 patients with CD and 411 patients with UC was established [3]. The patients either received or were considered candidates to anti-tumor necrosis factor-α (anti-TNF) therapy (infliximab or adalimumab) and were thus considered to be a homogenous group of moderately to severely ill IBD patients. The control group consisted of 795 healthy blood donors recruited from Viborg, Denmark [20].

### Genotyping

For the patients, DNA was extracted from cryopreserved blood clots by using the Maxwell 16 Blood purification kit (Promega, Madison, Wisconsin, USA) according to the manufacturers`instructions [21]. For the healthy controls, DNA was extracted from EDTA-stabilized peripheral blood by either PureGene (Qiagen, Hilden, Germany) or Wizard Genomic (Promega, Madison, Wisconsin, USA) DNA purification kit according to the manufacturers`instructions [20]. Competitive Allele-Specific Polymerase chain reaction (KASP^™^), an end-point PCR technology, was used by LGC Genomics for genotyping (LGC Genomics, Hoddesdon, United Kingdom) (http://www.lgcgenomics.com/).

The SNPs studied were *TLR1* (rs4833095), *TLR5* (rs5744174 and rs2072493), *TIRAP* (rs8177374), *CARD8* (rs2043211), *NLRP1* (rs878329 and rs2670660), *NLRP3* (rs10754558), *IL12B* (rs3212217 and rs6887695), *IL12RB1* (rs401502), *IL12RB2* (rs11810249), *IL17A* (rs8193036), *IL18* (rs1946518 and rs187238), *IFNGR1* (rs2234711), *IFNGR2* (rs8134145, rs8126756 and rs17882748), *TBX21* (rs17250932) and *JAK2* (rs12343867).

Genotyping of *TLR5* (rs2072493), *IFNGR2* (rs8134145) and *IL17A* (rs8193036) failed. The 18 SNPs were replicated in 94 randomly selected samples yielding >99% identical genotypes. The studied SNPs had minor allele frequencies of 0.09 to 0.50.

Linkage disequilibrium for the assessed SNPs in the same gene was calculated using the SNP Annotation and Proxy Search (SNAP) software [22]. In addition, linkage disequilibrium between the assessed SNPs in our study and known susceptibility loci in the same gene was calculated.

### Power calculations

The Genetic Power Calculator was utilized for power analysis of discrete traits [23]. The lowest minor allele frequency (MAF) of the studied SNPs was 0.14. The ‘high-risk allele frequency’ was set to 0.14, the ‘prevalence’ was set to 0.00241 (CD), 0.00263 (UC) and 0.00504 (IBD) [24], D-prime was set to 1, type I error rate was set to 0.05 and number of cases and control:case ratio was based on Table 1. This cohort study had more than 80% chance of detecting a dominant effect with an odds ratio (OR) of 1.4 for CD, 1.5 for UC and 1.4 for IBD.

**Table 1:** Description of the study participants.

	Crohns Disease (CD)	Ulcerative Colitis (UC)	Controls
	(n = 624)	(n = 411)	(n = 795)
**Gender: n (%)**			
Male	272 (44)	201 (49)	411 (52)
Female	352 (56)	210 (51)	384 (48)
**Age**:			
Median (5%-95%)	37 (20–67)	42 (20–72)	43 (23–60)
**Age at diagnosis**:			
Median (5%-95%)	25 (14–59)	33 (15–67)	-
**Smoking habits: n (%)**			
Smokers	178 (29)	30 (7)	207 (26)
Former smokers	64 (10)	86 (21)	392 (49)
Never smokers	156 (25)	102 (25)	189 (24)
Data not available	226 (36)	193 (47)	7 (1)
**Location UC: n (%)**			
Proctitis (E1)	-	53 (13)	-
Left side (E2)	-	183 (45)	-
Extensive (E3)	-	134 (33)	-
Data not available	-	41 (10)	-
**Location CD: n (%)**			
Colonic (L2)	208 (33)	-	-
Ileal (L1)	172 (28)	-	-
Ileocolonic (L3)	210 (34)	-	-
Data not available	34 (5)	-	-

### Statistical analysis

Logistic regression was used to compare genotype distributions among patients with CD, UC and IBD versus healthy controls. Odds ratio adjusted for age and gender were assessed (Table 2). Odds ratio unadjusted (crude) and adjusted for age, gender and smoking status were included as supplementary Tables (S2 and S3 Tables). A chi-square test was used to test for deviation from Hardy-Weinberg equilibrium among the healthy controls.

**Table 2:** Odds ratios (OR) (adjusted for age and sex) for genotypes studied among healthy controls and patients with Crohns disease (CD), ulcerative colitis (UC) and combined inflammatory bowel disease (IBD).

				Crohns disease (CD) ^1^			Ulcerative colitis (UC)^1^			Inflammatory bowel disease (IBD)^1^		
Gene (rs-number)	N_CD_	N_UC_	N_Control_	OR (95% CI)	p-value	Bonferroni corrected p-value	OR (95% CI)	p-value	Bonferroni corrected p-value	OR (95% CI)	p-value	Bonferroni corrected p-value
*TLR1* (rs4833095), MAF: 0.20												
TT	381	236	485									
TC	198	146	261	0.90 (0.67–1.20)	0.47	1.00	1.11 (0.82–1.51)	0.51	1.00	0.96 (0.76–1.22)	0.74	1.00
CC	41	25	20	3.15 (1.59–6.26)	0.001	0.02	2.92 (1.42–6.00)	0.004	0.07	2.96 (1.64–5.32)	0.0003	0.005
TC or CC	239	171	281	1.04 (0.78–1.37)	0.80	1.00	1.23 (0.92–1.65)	0.17	1.00	1.09 (0.87–1.37)	0.46	1.00
*TLR5* (rs5744174), MAF: 0.45												
TT	186	123	215									
TC	295	191	399	0.96 (0.70–1.33)	0.82	1.00	0.92 (0.66–1.30)	0.65	1.00	0.96 (0.73–1.25)	0.74	1.00
CC	141	95	144	1.54 (1.04–2.28)	0.03	0.63	1.24 (0.82–1.88)	0.30	1.00	1.36 (0.98–1.88)	0.07	1.00
TC or CC	436	286	543	1.10 (0.81–1.49)	0.54	1.00	1.01 (0.73–1.39)	0.96	1.00	1.06 (0.82–1.36)	0.65	1.00
*TIRAP* (rs8177374), MAF: 0.15												
CC	457	301	556									
CT	146	97	185	1.08 (0.79–1.48)	0.63	1.00	1.00 (0.72–1.41)	0.98	1.00	1.04 (0.80–1.36)	0.75	1.00
TT	16	8	21	0.86 (0.36–2.05)	0.73	1.00	0.68 (0.26–1.77)	0.43	1.00	0.74 (0.36–1.53)	0.42	1.00
CT or TT	162	105	206	1.06 (0.78–1.43)	0.71	1.00	0.97 (0.70–1.34)	0.84	1.00	1.01 (0.79–1.30)	0.93	1.00
*CARD8* (rs2043211), MAF: 0.35												
AA	305	182	321									
AT	246	175	342	0.80 (0.60–1.07)	0.13	1.00	0.94 (0.69–1.28)	0.69	1.00	0.82 (0.65–1.05)	0.11	1.00
TT	70	54	94	0.77 (0.50–1.20)	0.25	1.00	1.06 (0.68–1.65)	0.78	1.00	0.91 (0.64–1.30)	0.60	1.00
AT or TT	316	229	436	0.79 (0.61–1.04)	0.09	1.00	0.97 (0.73–1.29)	0.83	1.00	0.84 (0.67–1.06)	0.14	1.00
*NLRP1* (rs878329), MAF: 0.46												
GG	181	137	217									
GC	316	181	394	0.98 (0.72–1.35)	0.91	1.00	0.78 (0.56–1.09)	0.15	1.00	0.89 (0.68–1.15)	0.37	1.00
CC	122	91	155	0.87 (0.59–1.28)	0.47	1.00	1.03 (0.69–1.54)	0.89	1.00	1.00 (0.73–1.38)	0.99	1.00
GC or CC	438	272	549	0.95 (0.70–1.28)	0.72	1.00	0.85 (0.63–1.16)	0.32	1.00	0.92 (0.72–1.18)	0.51	1.00
*NLRP1* (rs2670660), MAF: 0.46												
AA	181	127	222									
AG	312	195	390	1.01 (0.74–1.39)	0.93	1.00	0.91 (0.65–1.27)	0.58	1.00	0.96 (0.74–1.24)	0.75	1.00
GG	128	87	154	0.92 (0.62–1.35)	0.65	1.00	1.05 (0.70–1.59)	0.80	1.00	1.03 (0.74–1.42)	0.87	1.00
AG or GG	440	282	544	0.98 (0.73–1.32)	0.91	1.00	0.95 (0.70–1.30)	0.75	1.00	0.98 (0.77–1.25)	0.86	1.00
*NLRP3* (rs10754558), MAF: 0.38												
CC	225	149	294									
CG	307	202	355	1.02 (0.76–1.37)	0.92	1.00	1.12 (0.82–1.53)	0.48	1.00	1.09 (0.85–1.39)	0.51	1.00
GG	84	58	111	0.95 (0.63–1.43)	0.80	1.00	1.14 (0.74–1.77)	0.55	1.00	1.08 (0.77–1.52)	0.66	1.00
CG or GG	391	260	466	1.00 (0.76–1.32)	0.99	1.00	1.13 (0.84–1.52)	0.44	1.00	1.08 (0.86–1.37)	0.50	1.00
*IL12B* (rs3212217), MAF: 0.19												
GG	402	287	499									
GC	194	106	235	0.89 (0.66–1.20)	0.44	1.00	0.77 (0.56–1.07)	0.12	1.00	0.81 (0.63–1.04)	0.10	1.00
CC	25	12	25	1.70 (0.82–3.51)	0.16	1.00	1.07 (0.46–2.50)	0.87	1.00	1.37 (0.73–2.56)	0.33	1.00
GC or CC	219	118	260	0.95 (0.72–1.26)	0.72	1.00	0.80 (0.58–1.09)	0.15	1.00	0.86 (0.67–1.09)	0.20	1.00
*IL12B* (rs6887695), MAF: 0.29												
GG	261	199	385									
GC	283	169	293	1.50 (1.12–2.00)	0.006	0.11	1.20 (0.88–1.64)	0.24	1.00	1.29 (1.01–1.64)	0.04	0.72
CC	71	39	72	1.45 (0.91–2.30)	0.11	1.00	1.19 (0.71–1.98)	0.51	1.00	1.29 (0.88–1.90)	0.20	1.00
GC or CC	354	208	365	1.49 (1.13–1.96)	0.004	0.08	1.20 (0.90–1.61)	0.22	1.00	1.29 (1.03–1.62)	0.03	0.52
*IL12RB1* (rs401502), MAF: 0.32												
CC	287	178	360									
CG	266	191	303	1.08 (0.81–1.44)	0.61	1.00	1.32 (0.97–1.78)	0.08	1.00	1.24 (0.98–1.58)	0.07	1.00
GG	67	39	87	0.92 (0.59–1.43)	0.70	1.00	0.93 (0.56–1.54)	0.78	1.00	0.91 (0.63–1.33)	0.64	1.00
CG or GG	333	230	390	1.04 (0.79–1.36)	0.78	1.00	1.23 (0.92–1.64)	0.16	1.00	1.17 (0.93–1.46)	0.18	1.00
*IL12RB2* (rs11810249), MAF: 0.00												
CC	622	408	774									
CT	1	2	0	1.00 (1.00–1.00)	1.00	1.00	1.00 (1.00–1.00)	1.00	1.00	1.00 (1.00–1.00)	1.00	1.00
TT	0	0	0	1.00 (1.00–1.00)	1.00	1.00	1.00 (1.00–1.00)	1.00	1.00	1.00 (1.00–1.00)	1.00	1.00
CT or TT	1	2	0	1.00 (1.00–1.00)	1.00	1.00	1.00 (1.00–1.00)	1.00	1.00	1.00 (1.00–1.00)	1.00	1.00
*IL18* (rs1946518), MAF: 0.39												
GG	236	148	282									
GT	299	201	363	1.03 (0.77–1.39)	0.84	1.00	1.05 (0.76–1.43)	0.78	1.00	1.05 (0.82–1.34)	0.68	1.00
TT	83	57	113	0.72 (0.47–1.11)	0.14	1.00	0.95 (0.61–1.49)	0.83	1.00	0.84 (0.59–1.19)	0.33	1.00
GT or TT	382	258	476	0.95 (0.72–1.26)	0.73	1.00	1.02 (0.76–1.38)	0.88	1.00	1.00 (0.79–1.26)	1.00	1.00
*IL18* (rs187238), MAF: 0.29												
GG	334	231	387									
GC	246	144	312	0.93 (0.70–1.23)	0.60	1.00	0.72 (0.53–0.98)	0.04	0.71	0.87 (0.69–1.11)	0.26	1.00
CC	36	32	64	0.50 (0.28–0.88)	0.02	0.31	0.73 (0.42–1.27)	0.26	1.00	0.61 (0.39–0.95)	0.03	0.57
GC or CC	282	176	376	0.85 (0.65–1.12)	0.26	1.00	0.72 (0.54–0.97)	0.03	0.54	0.83 (0.66–1.04)	0.10	1.00
*IFNGR1* (rs2234711), MAF: 0.39												
TT	235	158	290									
TC	276	196	361	0.89 (0.66–1.20)	0.44	1.00	1.18 (0.86–1.60)	0.31	1.00	1.03 (0.80–1.31)	0.83	1.00
CC	104	54	119	1.36 (0.91–2.02)	0.13	1.00	0.84 (0.52–1.34)	0.47	1.00	1.14 (0.81–1.60)	0.46	1.00
TC or CC	380	250	480	0.99 (0.75–1.32)	0.97	1.00	1.10 (0.81–1.48)	0.54	1.00	1.05 (0.84–1.33)	0.67	1.00
*IFNGR2* (rs8126756), MAF: 0.14												
TT	465	289	553									
TC	134	112	168	1.18 (0.85–1.64)	0.32	1.00	1.28 (0.91–1.79)	0.15	1.00	1.24 (0.94–1.62)	0.12	1.00
CC	13	3	18	0.67 (0.27–1.67)	0.39	1.00	0.33 (0.08–1.34)	0.12	1.00	0.55 (0.24–1.25)	0.15	1.00
TC or CC	147	115	186	1.12 (0.82–1.53)	0.49	1.00	1.18 (0.85–1.64)	0.33	1.00	1.16 (0.89–1.50)	0.27	1.00
*IFNGR2* (rs17882748), MAF: 0.47												
CC	180	113	199									
CT	295	214	391	0.83 (0.60–1.15)	0.26	1.00	0.98 (0.70–1.39)	0.92	1.00	0.90 (0.69–1.17)	0.43	1.00
TT	142	80	153	1.04 (0.71–1.52)	0.86	1.00	0.92 (0.60–1.40)	0.68	1.00	0.97 (0.70–1.34)	0.86	1.00
CT or TT	437	294	544	0.89 (0.66–1.21)	0.46	1.00	0.96 (0.69–1.34)	0.82	1.00	0.92 (0.71–1.18)	0.51	1.00
*TBX21* (rs17250932), MAF: 0.18												
TT	414	277	526									
TC	187	124	210	1.20 (0.89–1.61)	0.23	1.00	1.18 (0.86–1.61)	0.31	1.00	1.21 (0.94–1.55)	0.13	1.00
CC	18	10	32	0.62 (0.30–1.31)	0.21	1.00	0.64 (0.29–1.45)	0.29	1.00	0.65 (0.35–1.19)	0.16	1.00
TC or CC	205	134	242	1.11 (0.84–1.48)	0.46	1.00	1.10 (0.82–1.50)	0.52	1.00	1.13 (0.89–1.43)	0.31	1.00
*JAK2* (rs12343867), MAF: 0.28												
TT	294	204	398									
TC	264	166	299	1.19 (0.89–1.58)	0.23	1.00	1.21 (0.90–1.64)	0.21	1.00	1.21 (0.96–1.54)	0.11	1.00
CC	56	40	61	1.30 (0.79–2.14)	0.29	1.00	1.28 (0.75–2.16)	0.37	1.00	1.33 (0.88–2.01)	0.18	1.00
TC or CC	320	206	360	1.21 (0.92–1.59)	0.17	1.00	1.22 (0.92–1.63)	0.17	1.00	1.23 (0.98–1.54)	0.07	1.00

^1^Adjusted for age and sex. MAF: minor allele frequency.

Statistical analyses were performed using STATA version 11 (STATA Corp., Texas, USA).

### Ethics Statement

The study was conducted in accordance with the Declaration of Helsinki and was approved by the Regional Ethics Committees of Central (M20100153) and Southern (S-20120113) Denmark and the Danish Data Protection Agency of Central (RM: J. 2010-41-4719) and Southern (RSD: 2008-58-035) Denmark. The Ethics Committees gave suspension for obtaining written informed consent.

## Results

### Study population

Characteristics of the Danish patients with CD, UC and healthy controls are shown in Table 1. The genotype distributions among the healthy controls deviated from Hardy-Weinberg equilibrium for *TLR1* 743 T>C (rs4833095) (p = 0.03). After correction for multiple testing, all SNPs studied were in Hardy-Weinberg equilibrium.

### Polymorphisms associated with risk of CD

The homozygous variant genotype of *TLR1* 743 T>C (rs4833095) (OR: 3.15, 95% confidence interval (CI): 1.59–6.26, p = 0.001) and *TLR5* 936 T>C (rs5744174) (OR: 1.54, 95% CI: 1.04–2.28, p = 0.03) and the combined homozygous and the heterozygous variant genotypes of *IL12B* G>C (rs6887695) (OR: 1.49, 95% CI: 1.13–1.96, p = 0.004) were associated with increased risk of CD. The homozygous variant genotype of *IL18*–137 G>C (rs187238) (OR: 0.50, 95% CI: 0.28–0.88, p = 0.02) was associated with reduced risk of CD (Table 2).

After Bonferroni correction for multiple testing, the homozygous variant genotype of *TLR1* 743 T>C (rs4833095) (OR: 3.15, 95% CI: 1.59–6.26, p = 0.02) was associated with increased risk of CD.

### Polymorphisms associated with risk of UC

The homozygous variant genotype of *TLR1* 743 T>C (rs4833095) (OR: 2.92, 95% CI: 1.42–6.00, p = 0.004) was associated with increased risk of UC. The combined homozygous and the heterozygous variant genotypes of *IL18*–137 G>C (rs187238) (OR: 0.72, 95% CI: 0.54–0.97, p = 0.03) were associated with reduced risk of UC (Table 2).

### Polymorphisms associated with risk of IBD

In order to increase statistical power the analyses for CD and UC were combined (IBD). The studied polymorphisms generally showed the same direction of effect for CD and UC (Table 2).

The homozygous variant genotype of *TLR1* 743 T>C (rs4833095) (OR: 2.96, 95% CI: 1.64–5.32, p = 0.0003) and the combined homozygous and the heterozygous variant genotypes of *IL12B* G>C (rs6887695) (OR: 1.29, 95% CI: 1.03–1.62, p = 0.03) were associated with increased risk of IBD. The homozygous variant genotype of *IL18*–137 G>C (rs187238) (OR: 0.61, 95% CI: 0.39–0.95, p = 0.03) was associated with reduced risk of IBD (Table 2).

After Bonferroni correction for multiple testing, the homozygous variant genotype of *TLR1* 743 T>C (rs4833095) (OR: 2.96, 95% CI: 1.64–5.32, p = 0.005) was associated with increased risk of IBD.

The biologic effects of the studied SNPs and OR for polymorphisms which have been shown to be associated with risk of CD, UC or IBD in other studies and in this study were summarized in S1 Table.

### Linkage disequilibrium

Rs6887695 (*IL12B*) included in this study was in linkage disequilibrium with rs6556412 (*IL12B*) associated with CD in another study with r^2^ = 0.96 and D' = 1.00. No linkage disequilibrium was found for the SNPs in *TLR5* (rs5744174 and rs2072493), *NLRP1* (rs878329 and rs2670660), *IL12B* (rs3212217 and rs6887695), *IL18* (rs1946518 and rs187238) or *IFNGR2* (rs8134145, rs8126756 and rs17882748) (r^2^ < 0.8).

## Discussion

**Fig 1:**
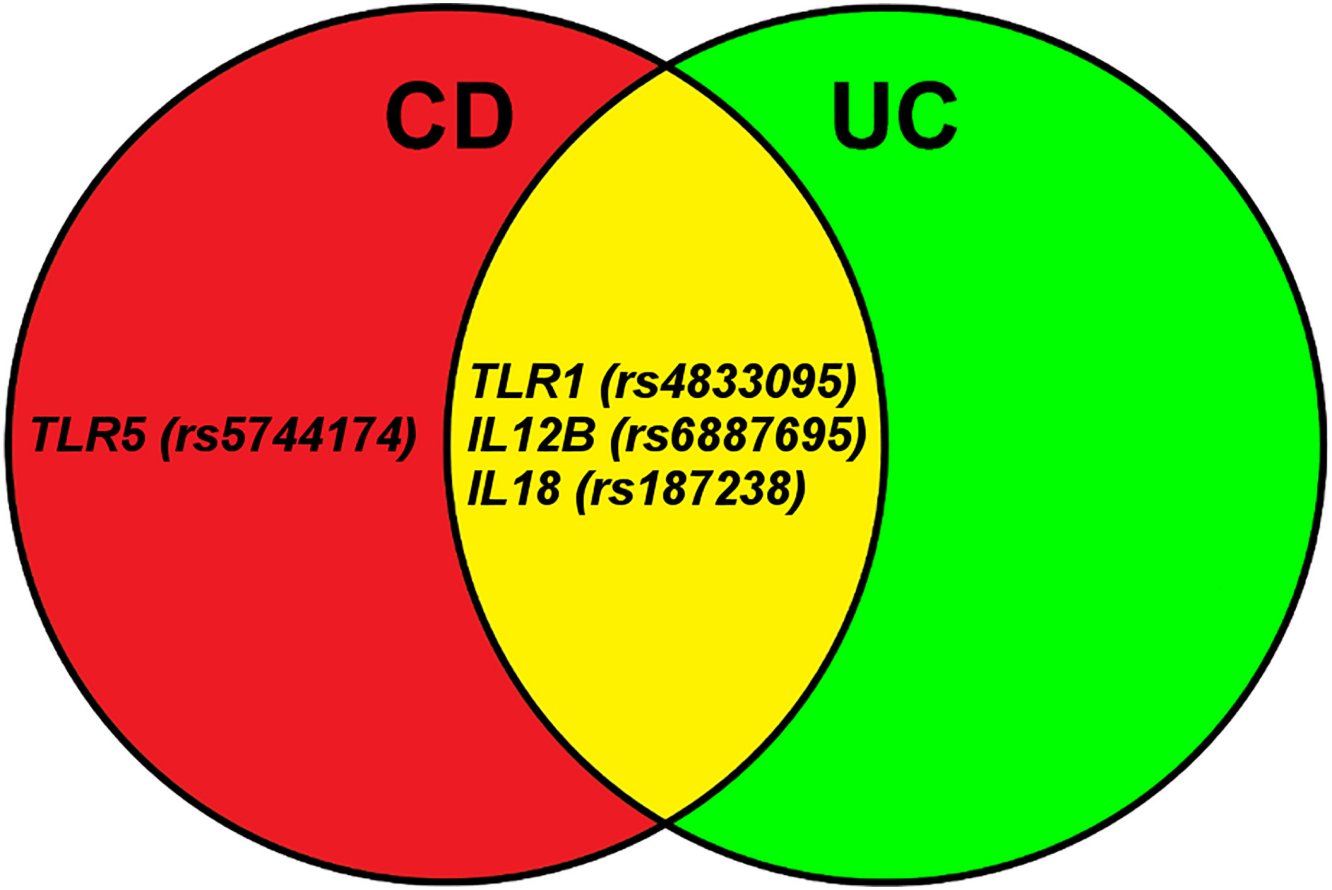
Eighteen functional single nucleotide polymorphisms (SNPs) in 14 genes were successfully genotyped and 4 SNPs in 4 genes were found to be associated with susceptability of severe Crohn’s disease (CD), ulcerative colitis (UC) or combined CD and UC. The genes are involved in the Toll-like receptor (*TLR1* and *TLR5*) and the IL-23/IL-17 (*IL12B* and *IL18*) pathways. Other studies have also found the *IL12B* G>C (rs6887695) polymorphism to be associated with risk of CD and our results confirm it as a risk locus. The *TLR1* 743 T>C (rs4833095)) polymorphism is a novel susceptibility loci.

In this Danish cohort of severely ill patients, 4 functional polymorphisms in 4 genes involved in the Toll-like receptor (*TLR1* and *TLR5*) and the IL-23/IL-17 (*IL12B* and *IL18*) pathways were found to be associated with risk of CD or UC (Fig 1).

In agreement with our study, the variant allele of the *IL12B* G>C (rs6887695) polymorphism has been associated with increased risk of CD, in the Welcome Trust Case Control Consortium and in a Japanese cohort [25,26]. Our study thereby confirms *IL12B* G>C (rs6887695) as a susceptibility locus for CD. *IL12B* encode the IL-12p40 subunit which is part of both the IL12 (IL-12p35/IL-12p40) and IL23 (IL-23p19/IL-12p40) heterodimers. All three subunits are up-regulated in patients with CD [27–30]. Furthermore, *IL12B* G>C (rs6887695) is in linkage disequilibrium with *IL12B* (rs6556412) which in another study has been associated with increased risk of CD [5] in agreement with the results in our study for rs6887695. In addition, the variant allele of *IL12B* G>C (rs6887695) has also been associated with increased risk of UC in a meta-analysis (OR: 1.11), and although the direction of association was the same in our study we were unable to confirm the result probably because of lack of power [31].

In contrast to our study, other case-control studies found opposite direction of association in other ethnic groups of patients with UC for *IL18*–137 G>C (rs187238) [32] or opposite direction of association in children with CD for *TLR5* 936 T>C (rs5744174) [33]. There is no consensus of how the stop codon polymorphism in *CARD8* A>T (rs2043211) influence risk of CD [34,35].

To the best of our knowledge the polymorphism in *TLR1* 743 T>C (rs4833095) found to be associated with both CD and UC in our study, has not previously been described as a susceptibility polymorphism.

The biological interpretation of our results indicates that genetically determined high activity of *TLR1* (rs4833095) was associated with increased risk of both CD and UC and genetically determined high activity of *TLR5* (rs5744174) was associated with increased risk of CD. Both TLR1 and TLR5 can activate NFκB, a central regulator of inflammation regulating the expression of more than 150 genes including *IL1B*, *IL12A* (p35), *IL12B* (p40), *IL17A*, *IL23A* (p19) and *IFNG* [36]. The associations found in *TLR1* and *TLR5* further supports that the host microbial composition or environmental factors in the gut are involved in risk of IBD in agreement with other studies [2,3].

The pro-protein IL-18 can be activated by the inflammasome and IL-18 can downstream induce the production of the pro-inflammatory cytokine IL-17 [10]. In our study, the associations found for the polymorphism -137 G>C (rs187238) indicates that genetically determined low activity of IL-18 was associated with reduced risk of CD and UC. This suggests that lower activity of the IL-23/IL-17 pathway was associated with reduced risk of CD and UC.

The activity of IL-17 can also be up-regulated through the IL-23 pathway and down-regulated through the IL-12 pathway. Many proteins involved in the IL-12 pathway are also involved in the IL-23 pathway, including IL-12p40 and Jak2. Our study indicates that genetically determined low IL-12p40 level (a subunit of both IL-12 and IL-23) was associated with increased risk of CD (rs6887695), in accordance with other studies [25,26]. It could be speculated, that a lower level of the IL-12p40 subunit results in a lower activity of the IL-12 pathway, lower inactivation of the pro-inflammatory cytokine IL-17 and thus increased inflammatory response, which might explain the increased risk of CD.

Overall, the polymorphism in *TLR5* (rs5744174) and *IL12B* (rs6887695) associated with CD, the polymorphism *TLR1* (rs4833095) and *IL18* (rs187238) associated with both CD and UC support that genetically determined high inflammatory response is associated with increased risk of both CD and UC. In addition, the polymorphism in *IL12B* (rs6887695) associated with CD and the polymorphism in *IL18* (rs187238) associated with both CD and UC suggest that genetically determined high activity of the IL-23/IL-17 pathway was associated with increased risk of CD and UC. It could be speculated that IL-17 plays a key role as IL-17 can be inhibited by the IL-12 pathway and induced by the IL-23 pathway.

Studies have shown that mice deficient in the IL-23 pathway specific subunit (IL-23p19) or subunits shared with IL12 (IL-12p40 and IL12R-β1) were resistant to inflammation, whereas mice deficient in IL-12 pathway specific subunits (IL-12p35 and IL12R-β2) were more susceptible to inflammation [14]. Antibodies targeting the shared subunit IL-12p40 (Ustekinumab) have been shown to be more effective than placebo in treating patients with severe CD, especially those who failed to respond to anti-TNF therapy [37]. Based on our results it could be speculated, that a better result might be obtained by using antibodies targeting the IL-23 specific subunit IL-23p19, as our study indicates a protective effect of IL-12p40 through the IL-12 pathway.

The results in this study should be interpreted with care. *TLR1* 743 T>C (rs4833095) was not in Hardy-Weinberg equilibrium among the healthy controls, which is probable due to chance. When corrected adequately for multiple testing no deviation from Hardy-Weinberg equilibrium was found. In the light of the obtained P-values and the number of statistical tests performed we cannot exclude, that some of our positive findings may be due to chance. If the results were corrected for multiple testing the homozygous variant genotype of *TLR1* 743 T>C (rs4833095) was associated with increased risk of CD (p = 0.02) and marginally with UC (p = 0.07) and the combined homozygous and the heterozygous variant genotypes of *IL12B* G>C (rs6887695) were marginally associated with increased risk of CD (p = 0.08). We successfully assessed 18 polymorphisms and assuming a 5% acceptance level, one polymorphism would be expected to be associated with susceptibility by pure chance. In this study 5 polymorphisms were found to be associated with susceptibility and most of the found associations were biologically plausible. We cannot exclude that associations were not identified due to insufficient statistical power. The results should therefore be replicated in independent cohorts. A major strength was that this clinically homogeneous and well-characterised cohort was rather large including 1035 patients with IBD and 795 healthy controls. All the patients were considered for anti-TNF treatment and were therefore considered to have a severe disease course. Genetic determinants may be expected to be strong among severely ill cases [38].

In conclusion, 4 functional SNPs in 4 genes involved in regulation of inflammation were found to be associated with susceptibility of severe CD or UC. The SNP in *TLR1* 743 (rs4833095) has not previously been reported as susceptibility polymorphisms of both CD and UC (Fig 1). The *IL12B* G>C (rs6887695) polymorphism associated with risk of CD in our study has also been associated with risk of CD in other studies and should therefore be considered a confirmed risk locus. Our results suggest that genetically determined high activity of *TLR1* and *TLR5* was associated with increased risk of CD and UC. This supports that the host microbial composition or environmental factors in the gut are involved in risk of IBD. Furthermore, genetically determined high activity of the IL-23/IL-17 pathway was associated with increased risk of CD and UC. Overall, our results support that genetically determined high inflammatory response was associated with increased risk of both CD and UC.

## Supporting Information

S1 Table The biologic effect of the studied single nucleotide polymorphism (SNP) and odds ratios (OR) for polymorphisms which have been shown to be associated with risk of Crohn's disease (CD), ulcerative colitis (UC) or inflammatory bowel disease (IBD) in other studies and in this study. (DOC)

S2 Table Odds ratios (OR) (unadjusted) for genotypes studied among healthy controls and patients with Crohns disease (CD), ulcerative colitis (UC) and combined inflammatory bowel disease (IBD). (DOC)

S3 Table Odds ratios (OR) (adjusted for age, sex and smoking status) for genotypes studied among healthy controls and patients with Crohns disease (CD), ulcerative colitis (UC) and combined inflammatory bowel disease (IBD). (DOC)
