# Supplementary material for: Polymorphisms in the Toll-Like Receptor and the IL-23/IL-17 Pathways Were Associated with Susceptibility to Inflammatory Bowel Disease in a Danish Cohort
Source: PLoS One. 2015 Dec 23;10(12):e0145302. doi: 10.1371/journal.pone.0145302 (PMC4689491; doi:10.1371/journal.pone.0145302)
Supplement: S1 Table — (DOC) [file pone.0145302.s001.doc]

**S1 Table:** The biologic effect of the studied single nucleotide polymorphism (SNP) and odds ratios (OR) for polymorphisms which have been shown to be associated with risk of Crohn's disease (CD), ulcerative colitis (UC) or inflammatory bowel disease (IBD) in other studies and in this study.

| Gene (SNP) | rs-number | Effect of the SNP | Associations found in other studies.  Disease, genotype, OR (95% CI), p-value | Associations found in this study.  Disease, genotype, OR (95% CI), p-value |
| --- | --- | --- | --- | --- |
| *TLR1* (activates inflammation through the canonical NFκB pathway) | | | | |
| 743 T>C | rs4833095 | 743C increases TLR1 level^A, C, E^  [1] | CD: T, p = 0.09 [2]  UC: C, 1.07 (1.02-1.12), p = 0.006 [2] | CD: CC: 3.15 (1.59-6.26), p = 0.001  UC: CC: 2.92 (1.42-6.00), p = 0.004  IBD: CC: 2.96 (1.64-5.32), p = 0.0003 |
|  | | | | |
| *TLR5* (activates inflammation through the canonical NFκB pathway) | | | | |
| 936 T>C | rs5744174 | 936C increases IFN-γ [3] and CCL20 level^E^ [4]; increases IL-6 and IL-1β mRNA level^D^[5];  936C reduce the risk of CD in children [4] | CD: C, 0.84 (0.71-1.00), p = 0.05 [4]  UC: G, 1.02 (0.97-1.06), p = 0.42 [2] | CD: CC: 1.54 (1.04-2.28), p = 0.03 |
|  | | | | |
| *TIRAP* (involved in TLR signalling) | | | | |
| C>T (S180L) | rs8177374 | 180L increases TNF-α, IL-6, IFN-γ level^E^ [6] | CD: C, p = 0.13 [2]  UC: T, 1.05 (0.99-1.11), p = 0.10 [2] | No association |
|  | | | | |
| *CARD8* (can suppress the activation of caspase-1 and thereby suppress both the apoptotic and the inflammasome pathway) | | | | |
| A>T (C10X) | rs2043211 | rs2043211T decrease expression^D^ [7]. CARD8-X associated with a worse disease cause in early rheumatoid arthritis (RA) [8] | CD: Inconclusiv [9]  UC: T, 1.06 (1.01-1.12), p = 0.02 [2] | No association |
|  | | | | |
| *NLRP1* (involved in activation of caspase-1 and caspase-5 as part of the NALP1 inflammasome complex, which leads to processing and release of IL-1β and IL-18. Stimulates apoptosis through activation of caspase-3) | | | | |
| G>C | rs878329 | rs878329C reduces mRNA level^A, B, D^.  GG associated with RA [10] | CD: Unknown  UC: Unknown | No association |
| A>G | rs2670660 | rs2670660G reduces transcription^A, D^ [11] | CD: Unknown  UC: Unknown | No association |
|  | | | | |
| *NLRP3* (involved in the apoptosis and the inflammasome complex which leads to processing and release of IL-1β and IL-18) | | | | |
| 29940 C>G | rs10754558 | 29940G increase expression and NALP3 mRNA staility^A, B, D^ [12] | CD: Unknown  UC: Unknown | No association |
|  | | | | |
| *IL12B* (interleukin-12p40 serves as a subunit of interleukin 12 and 23) | | | | |
| -10993 G>C | rs3212217 | -10993C increase IL-12 level^E^ [13] | CD: C, p = 0.0002 [2]  UC: C, 1.08 (1.01-1.15), p = 0.02 [2] | No association |
| G>C | rs6887695 | rs6887695C marginally associated with reduce level of IL-12p40^E^ [13,14] and increased risk of CD and UC [15] | CD: C, 1.26 (1.12-1.41) 9.21×10^-6^ [16]  UC: C, 1.11 (1.05-1.18), p = 0.0007 [2] | CD: GC or CC: 1.49 (1.13-1.96), p = 0.004  IBD: GC or CC: 1.29 (1.03-1.62), p = 0.03 |
|  | | | | |
| *IL12RB1* (a subunit shared by the interleukin 12 and 23 receptor) | | | | |
| 378 C>G | rs401502 | 378G reduce IL-12p40 level^C^ [14] | CD: Unknown  UC: Unknown | No association |
|  | | | | |
| *IL12RB2* (a subunit specific for the interleukin 12 receptor) | | | | |
| -237 C>T | rs11810249 | -237T reduces expression by 50%^B, D^ [17] | CD: Unknown  UC: Unknown | No association |
|  | | | | |
| *IL18* (a pro-inflammatory cytokine, feedback activates IFN-γ. Also known to enhance the production of IL-17, TNF-α and IL-1β) | | | | |
| -607 C>A | rs1946518 | -607AA reduces IL-18 level^E^ [18,19] and expression [20]. -607C & -137G increase transcription^A, B, D^ [21,22] | CD: T, p = 0.69 [2]  UC: A, 1.02 (0.97-1.06), p = 0.45 [2] | No association |
| -137 G>C | rs187238 | -137C reduces IL-18 level^E^ [19] and expression [20] | CD: Unknown  UC: Unknown | CD: CC: 0.50 (0.28-0.88), p = 0.02  UC: GC or CC: 0.72 (0.54-0.97), p= 0.03  IBD: CC: 0.61 (0.39-0.95), p = 0.03 |
|  | | | | |
| *IFNGR1* (the IFN-γ receptor is a heterodimer of IFNGR1 and IFNGR2) | | | | |
| -56 T>C | rs2234711 | -56T increases expression 10-fold^D^ [23,24] | CD: Unknown  UC: Unknown | No association |
|  | | | | |
| *IFNGR2* (the IFN-γ receptor is a heterodimer of IFNGR1 and IFNGR2) | | | | |
| A>C,  T>C,  T>C | rs8134145,  rs8126756,  rs17882748 | ATC haplotype increases expression^D^ [25] | CD: Unknown  UC: Unknown | No association |
|  | | | | |
| *TBX21* (transcription factor that controls the expression of IFN-γ) | | | | |
| -1514 T>C | rs17250932 | -1514C reduces expression, reduces T-bet (*TBX21*) and IFN-γ level and increases IL-4 level^B, C, D^ [26] | CD: Unknown  UC: Unknown | No association |
|  | | | | |
| *JAK2* (a kinase that interact with many different membrane receptors including the IFN-γ, IL12 and IL23 receptor) | | | | |
| T>C (V617F) | rs12343867 | rs12343867C reduces expression^A, B, D^ [27] | CD: Unknown  UC: Unknown | No association |

^A^ Function examined by reverse transcriptase PCR (RT-PCR)

^B^ Function examined by electrophoretic mobility shift assay (EMSA)

^C^ Function examined by flow cytometry

^D^ Function examined by luciferase reporter assay

^E^ Function examined by enzyme-linked immunosorbent assay (ELISA)

ND: not determined

References

1. Uciechowski P, Imhoff H, Lange C, Meyer CG, Browne EN, Kirsten DK, et al. (2011) Susceptibility to tuberculosis is associated with TLR1 polymorphisms resulting in a lack of TLR1 cell surface expression. J Leukoc Biol 90: 377-388.

2. <http://www.ibdgenetics.org/>

3. Dhiman N, Ovsyannikova IG, Vierkant RA, Ryan JE, Pankratz VS, Jacobson RM, et al. (2008) Associations between SNPs in toll-like receptors and related intracellular signaling molecules and immune responses to measles vaccine: preliminary results. Vaccine 26: 1731-1736.

4. Sheridan J, Mack DR, Amre DK, Israel DM, Cherkasov A, Li H, et al. (2013) A non-synonymous coding variant (L616F) in the TLR5 gene is potentially associated with Crohn's disease and influences responses to bacterial flagellin. PLoS One 8: e61326.

5. Klimosch SN, Forsti A, Eckert J, Knezevic J, Bevier M, Schonfels V, et al. (2013) Functional TLR5 genetic variants affect human colorectal cancer survival. Cancer Res . 0008-5472.

6. Ferwerda B, Alonso S, Banahan K, McCall MB, Giamarellos-Bourboulis EJ, Ramakers BP, et al. (2009) Functional and genetic evidence that the Mal/TIRAP allele variant 180L has been selected by providing protection against septic shock. Proc Natl Acad Sci U S A 106: 10272-10277.

7. Paramel GV, Folkersen L, Strawbridge RJ, Elmabsout AA, Sarndahl E, Lundman P, et al. (2013) CARD8 gene encoding a protein of innate immunity is expressed in human atherosclerosis and associated with markers of inflammation. Clin Sci (Lond) 125: 401-407.

8. Kastbom A, Johansson M, Verma D, Soderkvist P, Rantapaa-Dahlqvist S (2010) CARD8 p.C10X polymorphism is associated with inflammatory activity in early rheumatoid arthritis. Ann Rheum Dis 69: 723-726.

9. Fisher SA, Mirza MM, Onnie CM, Soars D, Lewis CM, Prescott NJ, et al. (2007) Combined evidence from three large British Association studies rejects TUCAN/CARD8 as an IBD susceptibility gene. Gastroenterology: 2078-2080.

10. Sui J, Li H, Fang Y, Liu Y, Li M, Zhong B, et al. (2012) NLRP1 gene polymorphism influences gene transcription and is a risk factor for rheumatoid arthritis in han chinese. Arthritis Rheum 64: 647-654.

11. Glinskii AB, Ma J, Ma S, Grant D, Lim CU, Sell S, et al. (2009) Identification of intergenic trans-regulatory RNAs containing a disease-linked SNP sequence and targeting cell cycle progression/differentiation pathways in multiple common human disorders. Cell Cycle 8: 3925-3942.

12. Hitomi Y, Ebisawa M, Tomikawa M, Imai T, Komata T, Hirota T, et al. (2009) Associations of functional NLRP3 polymorphisms with susceptibility to food-induced anaphylaxis and aspirin-induced asthma. J Allergy Clin Immunol 124: 779-785.

13. Wu JF, Wu TC, Chen CH, Ni YH, Chen HL, Hsu HY, et al. (2010) Serum levels of interleukin-10 and interleukin-12 predict early, spontaneous hepatitis B virus e antigen seroconversion. Gastroenterology 138: 165-172.

13. Eskandari-Nasab E, Moghadampour M, Asadi-Saghandi A, Kharazi-Nejad E, Rezaeifar A, Pourmasoumi H (2013) Levels of interleukin-(IL)-12p40 are markedly increased in Brucellosis among patients with specific IL-12B genotypes. Scand J Immunol 78: 85-91.

14. Tao YP, Wang WL, Li SY, Zhang J, Shi QZ, Zhao F, et al. (2012) Associations between polymorphisms in IL-12A, IL-12B, IL-12Rbeta1, IL-27 gene and serum levels of IL-12p40, IL-27p28 with esophageal cancer. J Cancer Res Clin Oncol 138: 1891-1900.

15. Glas J, Seiderer J, Wagner J, Olszak T, Fries C, Tillack C, et al. (2012) Analysis of IL12B gene variants in inflammatory bowel disease. PLoS One 7: e34349.

16. Parkes M, Barrett JC, Prescott NJ, Tremelling M, Anderson CA, Fisher SA, et al. (2007) Sequence variants in the autophagy gene IRGM and multiple other replicating loci contribute to Crohn's disease susceptibility. Nat Genet: 830-832.

17. Verma VK, Taneja V, Jaiswal A, Sharma S, Behera D, Sreenivas V, et al. (2012) Prevalence, distribution and functional significance of the -237C to T polymorphism in the IL-12Rbeta2 promoter in Indian tuberculosis patients. PLoS One 7: e34355.

18. Chen DY, Chen YM, Chen HH, Hsieh CW, Lin CC, Lan JL (2009) Functional association of interleukin 18 gene -607 (C/A) promoter polymorphisms with disease course in Chinese patients with adult-onset Still's disease. J Rheumatol 36: 2284-2289.

19. Jaiswal PK, Singh V, Srivastava P, Mittal RD (2013) Association of IL-12, IL-18 variants and serum IL-18 with bladder cancer susceptibility in North Indian population. Gene 519: 128-134.

20. Dziedziejko V, Kurzawski M, Paczkowska E, Machalinski B, Pawlik A (2012) The impact of IL18 gene polymorphisms on mRNA levels and interleukin-18 release by peripheral blood mononuclear cells. Postepy Hig Med Dosw 66: 409-414.

21. Giedraitis V, He B, Huang WX, Hillert J (2001) Cloning and mutation analysis of the human IL-18 promoter: a possible role of polymorphisms in expression regulation. J Neuroimmunol 112: 146-152.

22. Kim SH, Son JK, Yang EM, Kim JE, Park HS (2011) A functional promoter polymorphism of the human IL18 gene is associated with aspirin-induced urticaria. Br J Dermatol 165: 976-984.

23. Canedo P, Corso G, Pereira F, Lunet N, Suriano G, Figueiredo C, et al. (2008) The interferon gamma receptor 1 (IFNGR1) -56C/T gene polymorphism is associated with increased risk of early gastric carcinoma. Gut 57: 1504-1508.

24. Matsuda A, Ebihara N, Kumagai N, Fukuda K, Ebe K, Hirano K, et al. (2007) Genetic polymorphisms in the promoter of the interferon gamma receptor 1 gene are associated with atopic cataracts. Invest Ophthalmol Vis Sci 48: 583-589.

25. Hijikata M, Shojima J, Matsushita I, Tokunaga K, Ohashi J, Hang NT, et al. (2012) Association of IFNGR2 gene polymorphisms with pulmonary tuberculosis among the Vietnamese. Hum Genet 131: 675-682.

26. Li J, Li J, You Y, Chen S (2012) The role of upstream stimulatory factor 1 in the transcriptional regulation of the human TBX21 promoter mediated by the T-1514C polymorphism associated with systemic lupus erythematosus. Immunogenetics 64: 361-370.

27. Spasovski V, Tosic N, Nikcevic G, Stojiljkovic M, Zukic B, Radmilovic M, et al. (2013) The influence of novel transcriptional regulatory element in intron 14 on the expression of Janus kinase 2 gene in myeloproliferative neoplasms. J Appl Genet 54: 21-26.
