# Supplementary material for: Polymorphisms in the Toll-Like Receptor and the IL-23/IL-17 Pathways Were Associated with Susceptibility to Inflammatory Bowel Disease in a Danish Cohort
Source: PLoS One. 2015 Dec 23;10(12):e0145302. doi: 10.1371/journal.pone.0145302 (PMC4689491; doi:10.1371/journal.pone.0145302)
Supplement: S2 Table — (DOC) [file pone.0145302.s002.doc]

**S2 Table:** Odds ratios (OR) (unadjusted) for genotypes studied among healthy controls and patients with Crohns disease (CD), ulcerative colitis (UC) and combined inflammatory bowel disease (IBD).

|  | |  |  |  | **Crohns disease (CD)** ^1^ | | | **Ulcerative colitis (UC)**^1^ | | | **Inflammatory bowel disease (IBD)**^1^ | | |
| --- | --- | --- | --- | --- | --- | --- | --- | --- | --- | --- | --- | --- | --- |
| Gene (rs-number) | | **N_CD_** | **N_UC_** | **N_Control_** | OR (95% CI) | p-value | Bonferroni  corrected  p-value | OR (95% CI) | p-value | Bonferroni  corrected  p-value | OR (95% CI) | p-value | Bonferroni  corrected  p-value |
|  |  |  |  |  |  |  |  |  |  |  |  |  |  |
| *TLR1* (rs4833095), MAF: 0.20 | | | | | | | | | | | | | |
|  | TT | 381 | 236 | 485 |  |  |  |  |  |  |  |  |  |
|  | TC | 198 | 146 | 261 | 0.97 (0.77-1.21) | 0.76 | 1.00 | 1.15 (0.89-1.48) | 0.28 | 1.00 | 1.04 (0.85-1.27) | 0.73 | 1.00 |
|  | CC | 41 | 25 | 20 | 2.61 (1.50-4.53) | 0.0006 | 0.01 | 2.57 (1.40-4.72) | 0.002 | 0.04 | 2.59 (1.55-4.33) | 0.0003 | 0.006 |
|  | TC or CC | 239 | 171 | 281 | 1.08 (0.87-1.35) | 0.48 | 1.00 | 1.25 (0.98-1.60) | 0.07 | 1.00 | 1.15 (0.95-1.39) | 0.16 | 1.00 |
|  |  |  |  |  |  |  |  |  |  |  |  |  |  |
| *TLR5* (rs5744174), MAF: 0.45 | | | | | | | | | | | | | |
|  | TT | 186 | 123 | 215 |  |  |  |  |  |  |  |  |  |
|  | TC | 295 | 191 | 399 | 0.85 (0.67-1.09) | 0.21 | 1.00 | 0.84 (0.63-1.11) | 0.21 | 1.00 | 0.85 (0.68-1.05) | 0.14 | 1.00 |
|  | CC | 141 | 95 | 144 | 1.13 (0.84-1.53) | 0.42 | 1.00 | 1.15 (0.82-1.62) | 0.41 | 1.00 | 1.14 (0.87-1.49) | 0.34 | 1.00 |
|  | TC or CC | 436 | 286 | 543 | 0.93 (0.74-1.17) | 0.53 | 1.00 | 0.92 (0.71-1.20) | 0.54 | 1.00 | 0.93 (0.75-1.14) | 0.46 | 1.00 |
|  |  |  |  |  |  |  |  |  |  |  |  |  |  |
| *TIRAP* (rs8177374), MAF: 0.15 | | | | | | | | | | | | | |
|  | CC | 457 | 301 | 556 |  |  |  |  |  |  |  |  |  |
|  | CT | 146 | 97 | 185 | 0.96 (0.75-1.23) | 0.75 | 1.00 | 0.97 (0.73-1.29) | 0.82 | 1.00 | 0.96 (0.77-1.20) | 0.74 | 1.00 |
|  | TT | 16 | 8 | 21 | 0.93 (0.48-1.80) | 0.82 | 1.00 | 0.70 (0.31-1.61) | 0.40 | 1.00 | 0.84 (0.46-1.52) | 0.56 | 1.00 |
|  | CT or TT | 162 | 105 | 206 | 0.96 (0.75-1.22) | 0.72 | 1.00 | 0.94 (0.72-1.24) | 0.67 | 1.00 | 0.95 (0.77-1.18) | 0.64 | 1.00 |
|  |  |  |  |  |  |  |  |  |  |  |  |  |  |
| *CARD8* (rs2043211), MAF: 0.35 | | | | | | | | | | | | | |
|  | AA | 305 | 182 | 321 |  |  |  |  |  |  |  |  |  |
|  | AT | 246 | 175 | 342 | 0.76 (0.60-0.95) | 0.02 | 0.38 | 0.90 (0.70-1.17) | 0.43 | 1.00 | 0.81 (0.66-0.99) | 0.04 | 0.76 |
|  | TT | 70 | 54 | 94 | 0.78 (0.55-1.11) | 0.17 | 1.00 | 1.01 (0.69-1.48) | 0.95 | 1.00 | 0.87 (0.64-1.18) | 0.37 | 1.00 |
|  | AT or TT | 316 | 229 | 436 | 0.76 (0.62-0.94) | 0.01 | 0.19 | 0.93 (0.73-1.18) | 0.54 | 1.00 | 0.82 (0.68-1.00) | 0.04 | 0.76 |
|  |  |  |  |  |  |  |  |  |  |  |  |  |  |
| *NLRP1* (rs878329), MAF: 0.46 | | | | | | | | | | | | | |
|  | GG | 181 | 137 | 217 |  |  |  |  |  |  |  |  |  |
|  | GC | 316 | 181 | 394 | 0.96 (0.75-1.23) | 0.76 | 1.00 | 0.73 (0.55-0.96) | 0.02 | 0.38 | 0.86 (0.69-1.07) | 0.18 | 1.00 |
|  | CC | 122 | 91 | 155 | 0.94 (0.69-1.28) | 0.71 | 1.00 | 0.93 (0.66-1.30) | 0.67 | 1.00 | 0.94 (0.72-1.23) | 0.64 | 1.00 |
|  | GC or CC | 438 | 272 | 549 | 0.96 (0.76-1.21) | 0.71 | 1.00 | 0.78 (0.61-1.02) | 0.07 | 1.00 | 0.88 (0.72-1.08) | 0.23 | 1.00 |
|  |  |  |  |  |  |  |  |  |  |  |  |  |  |
| *NLRP1* (rs2670660), MAF: 0.46 | | | | | | | | | | | | | |
|  | AA | 181 | 127 | 222 |  |  |  |  |  |  |  |  |  |
|  | AG | 312 | 195 | 390 | 0.98 (0.77-1.26) | 0.88 | 1.00 | 0.87 (0.66-1.15) | 0.34 | 1.00 | 0.94 (0.75-1.16) | 0.56 | 1.00 |
|  | GG | 128 | 87 | 154 | 1.02 (0.75-1.38) | 0.90 | 1.00 | 0.99 (0.70-1.39) | 0.94 | 1.00 | 1.01 (0.77-1.32) | 0.96 | 1.00 |
|  | AG or GG | 440 | 282 | 544 | 0.99 (0.79-1.25) | 0.95 | 1.00 | 0.91 (0.70-1.18) | 0.46 | 1.00 | 0.96 (0.78-1.17) | 0.67 | 1.00 |
|  |  |  |  |  |  |  |  |  |  |  |  |  |  |
| *NLRP3* (rs10754558), MAF: 0.38 | | | | | | | | | | | | | |
|  | CC | 225 | 149 | 294 |  |  |  |  |  |  |  |  |  |
|  | CG | 307 | 202 | 355 | 1.13 (0.90-1.42) | 0.30 | 1.00 | 1.12 (0.86-1.46) | 0.39 | 1.00 | 1.13 (0.92-1.38) | 0.25 | 1.00 |
|  | GG | 84 | 58 | 111 | 0.99 (0.71-1.38) | 0.95 | 1.00 | 1.03 (0.71-1.50) | 0.87 | 1.00 | 1.01 (0.75-1.35) | 0.97 | 1.00 |
|  | CG or GG | 391 | 260 | 466 | 1.10 (0.88-1.37) | 0.41 | 1.00 | 1.10 (0.86-1.41) | 0.45 | 1.00 | 1.10 (0.90-1.33) | 0.34 | 1.00 |
|  |  |  |  |  |  |  |  |  |  |  |  |  |  |
| *IL12B* (rs3212217), MAF: 0.19 | | | | | | | | | | | | | |
|  | GG | 402 | 287 | 499 |  |  |  |  |  |  |  |  |  |
|  | GC | 194 | 106 | 235 | 1.02 (0.81-1.29) | 0.84 | 1.00 | 0.78 (0.60-1.03) | 0.08 | 1.00 | 0.92 (0.75-1.14) | 0.46 | 1.00 |
|  | CC | 25 | 12 | 25 | 1.24 (0.70-2.19) | 0.46 | 1.00 | 0.83 (0.41-1.68) | 0.61 | 1.00 | 1.07 (0.64-1.80) | 0.79 | 1.00 |
|  | GC or CC | 219 | 118 | 260 | 1.05 (0.84-1.31) | 0.69 | 1.00 | 0.79 (0.61-1.02) | 0.08 | 1.00 | 0.94 (0.77-1.14) | 0.53 | 1.00 |
|  |  |  |  |  |  |  |  |  |  |  |  |  |  |
| *IL12B* (rs6887695), MAF: 0.29 | | | | | | | | | | | | | |
|  | GG | 261 | 199 | 385 |  |  |  |  |  |  |  |  |  |
|  | GC | 283 | 169 | 293 | 1.42 (1.14-1.79) | 0.002 | 0.04 | 1.12 (0.86-1.44) | 0.40 | 1.00 | 1.29 (1.06-1.58) | 0.01 | 0.18 |
|  | CC | 71 | 39 | 72 | 1.45 (1.01-2.09) | 0.04 | 0.76 | 1.05 (0.68-1.60) | 0.83 | 1.00 | 1.28 (0.92-1.77) | 0.14 | 1.00 |
|  | GC or CC | 354 | 208 | 365 | 1.43 (1.15-1.77) | 0.001 | 0.02 | 1.10 (0.87-1.40) | 0.43 | 1.00 | 1.29 (1.07-1.56) | 0.009 | 0.17 |
|  |  |  |  |  |  |  |  |  |  |  |  |  |  |
| *IL12RB1* (rs401502), MAF: 0.32 | | | | | | | | | | | | | |
|  | CC | 287 | 178 | 360 |  |  |  |  |  |  |  |  |  |
|  | CG | 266 | 191 | 303 | 1.10 (0.88-1.38) | 0.40 | 1.00 | 1.27 (0.99-1.65) | 0.06 | 1.00 | 1.17 (0.96-1.43) | 0.13 | 1.00 |
|  | GG | 67 | 39 | 87 | 0.97 (0.67-1.38) | 0.85 | 1.00 | 0.91 (0.60-1.38) | 0.65 | 1.00 | 0.94 (0.69-1.29) | 0.72 | 1.00 |
|  | CG or GG | 333 | 230 | 390 | 1.07 (0.87-1.33) | 0.53 | 1.00 | 1.19 (0.94-1.52) | 0.15 | 1.00 | 1.12 (0.93-1.35) | 0.25 | 1.00 |
|  |  |  |  |  |  |  |  |  |  |  |  |  |  |
| *IL12RB2* (rs11810249), MAF: 0.00 | | | | | | | | | | | | | |
|  | CC | 622 | 408 | 774 |  |  |  |  |  |  |  |  |  |
|  | CT | 1 | 2 | 0 | 1.00 (1.00-1.00) | 1.00 | 1.00 | 1.00 (1.00-1.00) | 1.00 | 1.00 | 1.00 (1.00-1.00) | 1.00 | 1.00 |
|  | TT | 0 | 0 | 0 | 1.00 (1.00-1.00) | 1.00 | 1.00 | 1.00 (1.00-1.00) | 1.00 | 1.00 | 1.00 (1.00-1.00) | 1.00 | 1.00 |
|  | CT or TT | 1 | 2 | 0 | 1.00 (1.00-1.00) | 1.00 | 1.00 | 1.00 (1.00-1.00) | 1.00 | 1.00 | 1.00 (1.00-1.00) | 1.00 | 1.00 |
|  |  |  |  |  |  |  |  |  |  |  |  |  |  |
| *IL18* (rs1946518), MAF: 0.39 | | | | | | | | | | | | | |
|  | GG | 236 | 148 | 282 |  |  |  |  |  |  |  |  |  |
|  | GT | 299 | 201 | 363 | 0.98 (0.78-1.24) | 0.89 | 1.00 | 1.06 (0.81-1.37) | 0.69 | 1.00 | 1.01 (0.82-1.24) | 0.91 | 1.00 |
|  | TT | 83 | 57 | 113 | 0.88 (0.63-1.22) | 0.44 | 1.00 | 0.96 (0.66-1.40) | 0.84 | 1.00 | 0.91 (0.68-1.22) | 0.53 | 1.00 |
|  | GT or TT | 382 | 258 | 476 | 0.96 (0.77-1.19) | 0.71 | 1.00 | 1.03 (0.80-1.33) | 0.80 | 1.00 | 0.99 (0.81-1.20) | 0.90 | 1.00 |
|  |  |  |  |  |  |  |  |  |  |  |  |  |  |
| *IL18* (rs187238), MAF: 0.29 | | | | | | | | | | | | | |
|  | GG | 334 | 231 | 387 |  |  |  |  |  |  |  |  |  |
|  | GC | 246 | 144 | 312 | 0.91 (0.73-1.14) | 0.43 | 1.00 | 0.77 (0.60-1.00) | 0.05 | 0.95 | 0.86 (0.70-1.04) | 0.12 | 1.00 |
|  | CC | 36 | 32 | 64 | 0.65 (0.42-1.01) | 0.05 | 0.95 | 0.84 (0.53-1.32) | 0.45 | 1.00 | 0.73 (0.51-1.05) | 0.09 | 1.00 |
|  | GC or CC | 282 | 176 | 376 | 0.87 (0.70-1.08) | 0.20 | 1.00 | 0.78 (0.62-1.00) | 0.05 | 0.95 | 0.83 (0.69-1.01) | 0.06 | 1.00 |
|  |  |  |  |  |  |  |  |  |  |  |  |  |  |
| *IFNGR1* (rs2234711), MAF: 0.39 | | | | | | | | | | | | | |
|  | TT | 235 | 158 | 290 |  |  |  |  |  |  |  |  |  |
|  | TC | 276 | 196 | 361 | 0.94 (0.75-1.19) | 0.62 | 1.00 | 1.00 (0.77-1.29) | 0.98 | 1.00 | 0.96 (0.79-1.18) | 0.73 | 1.00 |
|  | CC | 104 | 54 | 119 | 1.08 (0.79-1.48) | 0.64 | 1.00 | 0.83 (0.57-1.21) | 0.34 | 1.00 | 0.98 (0.74-1.30) | 0.89 | 1.00 |
|  | TC or CC | 380 | 250 | 480 | 0.98 (0.79-1.22) | 0.83 | 1.00 | 0.96 (0.75-1.22) | 0.72 | 1.00 | 0.97 (0.80-1.17) | 0.74 | 1.00 |
|  |  |  |  |  |  |  |  |  |  |  |  |  |  |
| *IFNGR2* (rs8126756), MAF: 0.14 | | | | | | | | | | | | | |
|  | TT | 465 | 289 | 553 |  |  |  |  |  |  |  |  |  |
|  | TC | 134 | 112 | 168 | 0.95 (0.73-1.23) | 0.69 | 1.00 | 1.28 (0.97-1.68) | 0.09 | 1.00 | 1.07 (0.86-1.34) | 0.53 | 1.00 |
|  | CC | 13 | 3 | 18 | 0.86 (0.42-1.77) | 0.68 | 1.00 | 0.32 (0.09-1.09) | 0.07 | 1.00 | 0.65 (0.33-1.29) | 0.22 | 1.00 |
|  | TC or CC | 147 | 115 | 186 | 0.94 (0.73-1.21) | 0.63 | 1.00 | 1.18 (0.90-1.55) | 0.23 | 1.00 | 1.03 (0.83-1.28) | 0.77 | 1.00 |
|  |  |  |  |  |  |  |  |  |  |  |  |  |  |
| *IFNGR2* (rs17882748), MAF: 0.47 | | | | | | | | | | | | | |
|  | CC | 180 | 113 | 199 |  |  |  |  |  |  |  |  |  |
|  | CT | 295 | 214 | 391 | 0.83 (0.65-1.07) | 0.16 | 1.00 | 0.96 (0.73-1.28) | 0.80 | 1.00 | 0.88 (0.71-1.11) | 0.28 | 1.00 |
|  | TT | 142 | 80 | 153 | 1.03 (0.76-1.39) | 0.87 | 1.00 | 0.92 (0.65-1.31) | 0.65 | 1.00 | 0.99 (0.75-1.30) | 0.92 | 1.00 |
|  | CT or TT | 437 | 294 | 544 | 0.89 (0.70-1.13) | 0.33 | 1.00 | 0.95 (0.73-1.25) | 0.72 | 1.00 | 0.91 (0.74-1.13) | 0.40 | 1.00 |
|  |  |  |  |  |  |  |  |  |  |  |  |  |  |
| *TBX21* (rs17250932), MAF: 0.18 | | | | | | | | | | | | | |
|  | TT | 414 | 277 | 526 |  |  |  |  |  |  |  |  |  |
|  | TC | 187 | 124 | 210 | 1.13 (0.89-1.43) | 0.30 | 1.00 | 1.12 (0.86-1.46) | 0.40 | 1.00 | 1.13 (0.92-1.39) | 0.26 | 1.00 |
|  | CC | 18 | 10 | 32 | 0.71 (0.40-1.29) | 0.27 | 1.00 | 0.59 (0.29-1.23) | 0.16 | 1.00 | 0.67 (0.40-1.12) | 0.13 | 1.00 |
|  | TC or CC | 205 | 134 | 242 | 1.08 (0.86-1.35) | 0.52 | 1.00 | 1.05 (0.81-1.36) | 0.70 | 1.00 | 1.07 (0.87-1.30) | 0.53 | 1.00 |
|  |  |  |  |  |  |  |  |  |  |  |  |  |  |
| *JAK2* (rs12343867), MAF: 0.28 | | | | | | | | | | | | | |
|  | TT | 294 | 204 | 398 |  |  |  |  |  |  |  |  |  |
|  | TC | 264 | 166 | 299 | 1.20 (0.96-1.50) | 0.12 | 1.00 | 1.08 (0.84-1.40) | 0.54 | 1.00 | 1.15 (0.94-1.40) | 0.17 | 1.00 |
|  | CC | 56 | 40 | 61 | 1.24 (0.84-1.84) | 0.28 | 1.00 | 1.28 (0.83-1.97) | 0.26 | 1.00 | 1.26 (0.89-1.78) | 0.20 | 1.00 |
|  | TC or CC | 320 | 206 | 360 | 1.20 (0.97-1.49) | 0.09 | 1.00 | 1.11 (0.88-1.42) | 0.37 | 1.00 | 1.17 (0.97-1.41) | 0.11 | 1.00 |

^1^Crude (unadjusted). MAF: minor allele frequency.
